# *In vivo* assembly to switch fluorescent protein expression in *Escherichia coli*: a simple laboratory class to introduce molecular cloning

**DOI:** 10.1128/jmbe.00089-26

**Published:** 2026-07-15

**Authors:** Christopher J. Roberts, Kevin Gaston, Padma-Sheela Jayaraman

**Affiliations:** 1 Biodiscovery Institute, School of Medicine, University of Nottingham, Nottingham, United Kingdom; Charles Sturt University, Wagga Wagga, New South Wales, Australia

**Keywords:** molecular cloning, *in vivo* assembly, genetic recombination, fluorescence

## Abstract

Molecular cloning is a fundamental technique in life science research. However, learning to perform traditional restriction-ligation-based methods is time-consuming and prone to failure, discouraging new students. Here, we describe a compact, two- to three-session undergraduate practical class using *In vivo* Assembly, a simple, homology-based method that takes advantage of the native recombination machinery of *Escherichia coli*. Students swap a green fluorescent protein coding sequence with a red fluorescent protein coding sequence in a bacterial expression vector using PCR-amplified DNA fragments with short homology arms, followed by DpnI restriction enzyme digestion to remove template DNA, and then bacterial transformation. Success is directly visible by red versus green fluorescent bacterial colonies, providing rapid feedback and high student engagement. This low-cost protocol introduces key cloning concepts (PCR, homology design, template removal, bacterial transformation, and recombination) with reduced hands-on time and fewer failure points than classic approaches, making it ideal for effective teaching in resource-limited settings.

## INTRODUCTION

Molecular cloning underpins modern genetic engineering, synthetic biology, and biotechnology and is thus often taught in undergraduate biology and microbiology courses (1). Traditional restriction enzyme-based cloning, while conceptually simple, is labor-intensive, requiring agarose gel-based DNA purification and DNA ligation, and often yields low success rates for beginners (2). Frequent failures can undermine student confidence and motivation (3–5).

Homology-based assembly methods (e.g., Gibson and In-Fusion) enable scarless, multi-fragment DNA cloning. As cloning technologies have advanced, educators have recognized the need to update cloning curricula to reflect contemporary research practices(6). However, these techniques require specialized kits, multiple enzymes, or non-standard host organisms, which can introduce barriers to implementation, particularly in teaching laboratories with limited resources.

*In vivo* Assembly (IVA), first described in 1991 (7) but recently re-emerging (8), provides an effective alternative. IVA uses the RecA-independent recombination (RAIR) pathway in standard *Escherichia coli* cloning strains (e.g., DH5α, TOP10) to assemble linear DNA fragments with short terminal homology arms (15–40 bp) directly inside the cell upon transformation. Though the exact biochemistry of RAIR is not fully elucidated, a proposed model (8) involves *in vivo* resection of the DNA ends by 5′ to 3′ exonucleases to generate single-stranded DNA, which is then bound by RarA protein to facilitate annealing between the homologous sequences (9), followed by polymerase-mediated gap filling and finally DNA backbone ligation.

This article presents a ready-to-implement practical class in which students use IVA to replace an enhanced green fluorescent protein (eGFP) coding sequence (CDS) in a bacterial expression plasmid for an mCherry fluorescent protein CDS. This produces green (background) and red (successful assembly) fluorescent bacterial colonies. While we used fluorescent protein CDSs and bacterial expression here for easy readout of success, IVA is readily adaptable for many cloning procedures (8), and we have successfully used this technique to clone numerous CDSs into a variety of vectors.

Key benefits of this practical class:

- Short hands-on time and few failure points; no gel purification or *in vitro* ligation needed.
- An introduction to contemporary seamless cloning at low reagent cost.
- Built-in visual readout of success via fluorescent protein switching.

### Target audience and prerequisites

This class is suitable for first or second-year undergraduate students in any biological science program. No prior molecular cloning experience is needed. Basic micro-pipetting skills are required. Students will learn microbiology techniques such as aseptic technique, plate pouring, and bacterial transformation during the class itself. This class could also potentially be useful for consolidating learning about mechanisms of DNA repair. We successfully ran this class with second-year undergraduate students at the University of Nottingham, UK.

## MATERIALS

### Strains and plasmids

- Chemically competent *E. coli* DH5α (or equivalent RecA-deficient strain). These can be prepared in-house, e.g., by the CCMB80 method (10) for cost-saving and easy attainment of >10^7^ CFU/μg competency. Alternatively, they can be purchased.
- Template plasmid: pCR4-eGFP (eGFP under *lac* promoter; prepared in a Dam+ bacterial strain for DpnI sensitivity; construction details in the supplemental material).
- Donor plasmid: a plasmid containing mCherry CDS and lacking ampicillin resistance marker and lacking a bacterial promoter driving bacterial expression of mCherry, such as pmCherry-C1 mCherry-NLS (Addgene #58476).

### Reagents

- Phusion high-fidelity DNA polymerase (New England Biolabs) with HF buffer.
- DpnI restriction enzyme.
- Agarose, DNA ladder, gel stain, and electrophoresis buffer.
- LB agar, ampicillin, and Petri dishes.
- Standard molecular biology consumables (Eppendorf tubes, tips, etc.).

### Primers

See Table 1 for primer details.

**TABLE 1:** Sequences of PCR primers used by students for amplification of the pCR4 plasmid backbone and the mCherry insert^*a*^

Name	Sequence (5′–3′)
pCR4 backbone forward	CGGCCGCTAAATTCAATTCG
pCR4 backbone reverse	GTTTAAACCTGCAGGACTAGTCC
mCherry forward	AGGGACTAGTCCTGCAGGTTTAAACGTGAGCAAGGGCGAGG
mCherry reverse	TAGGGCGAATTGAATTTAGCGGCCGTTCATTACTTGTACAGCTCGTCC

^*a*^ The 25 bp homology arms incorporated in the mCherry primers are underlined. These should be provided to students at 10 μM in ddH_2_O.

### Equipment

A thermal cycler, microcentrifuge, micropipettes, agarose gel electrophoresis setup, gel imager, 37°C incubator, and Bunsen burner were used. Red coloration of colonies can easily be discerned in daylight, but a blue light and/or green light transilluminator can be used to enhance visualization.

### Safety

Follow standard Biosafety Level 1 practices for *E. coli* DH5α (Risk Group 1). Guidelines for Biosafety in Teaching Laboratories can be found here (11). Decontaminate all waste by autoclaving or disinfectant. Ensure appropriate shielding and tinted filters are in place when using transilluminators to avoid retinal damage.

## PROCEDURE

**Fig 1:**
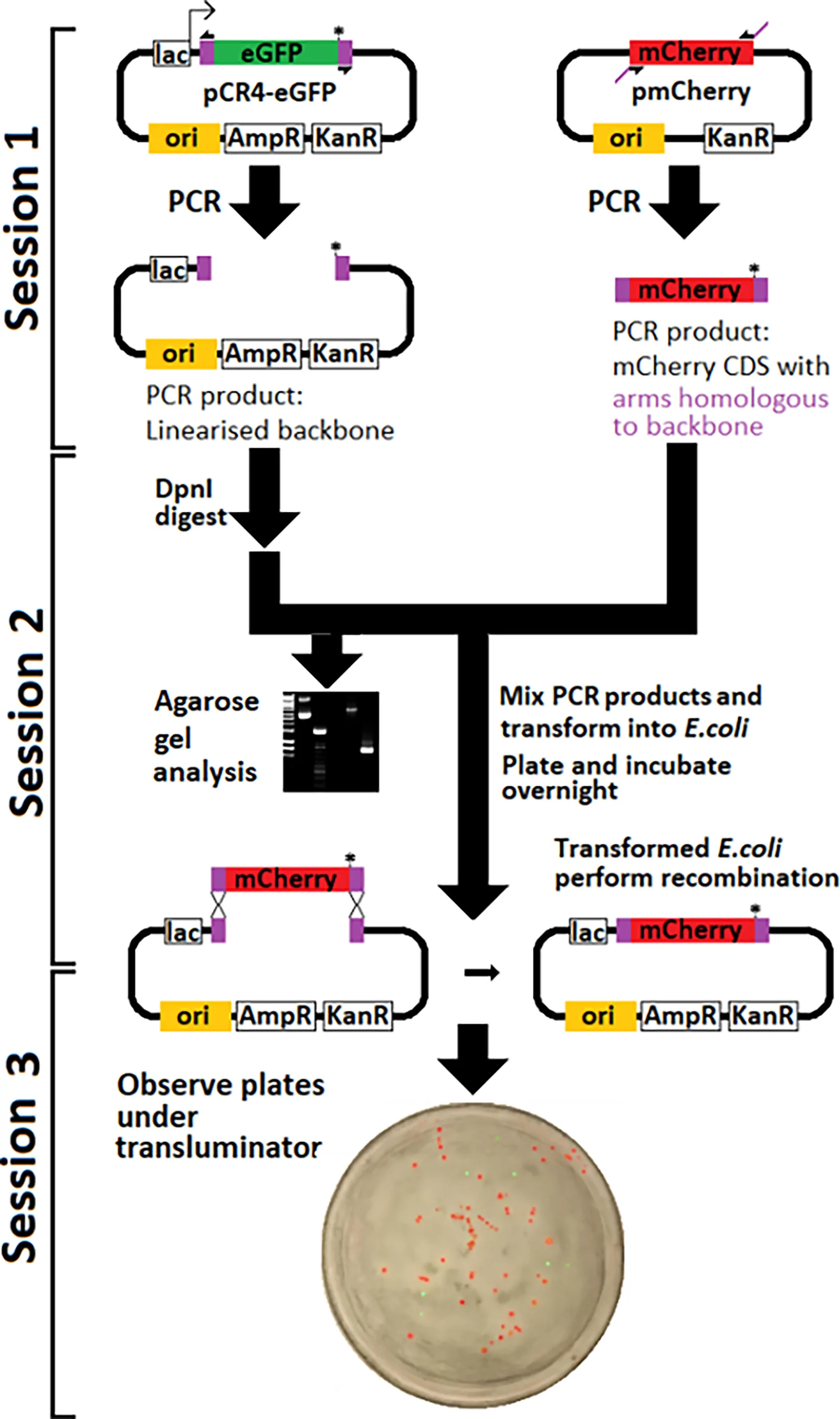
Workflow of the IVA practical class. Session 1: PCR amplification of pCR4 backbone and mCherry insert with homology arms. Session 2: DpnI digestion, agarose gel analysis, and bacterial transformation. Session 3: overnight incubation and colony scoring under daylight/blue light/green light.

The class spans three short sessions (two half-days or one full day and one brief observation session). Students use PCR to amplify the vector backbone (excluding the eGFP CDS) and mCherry CDS with added homology arms, digest the template DNA with DpnI, co-transform bacteria, and spread onto agar plates, and then score the resulting red/green bacterial colonies (Fig. 1).

### Session 1: PCR amplification (half-day)

Students set up two 25 μL PCRs using Phusion polymerase with HF buffer according to the manufacturer’s protocol, with the PCR primers listed in Table 1 and 50 ng of template DNA. The PCR should be performed under the following cycling conditions: 30 cycles—melting at 98°C for 15 s, annealing at 62°C for 15 s, and extension at 72°C for 2 min.

### Session 2: template digestion, analysis, and transformation (half-day)

Students set aside a 5 μL aliquot of each PCR product for gel analysis.

Students then set up restriction digests of the backbone PCR product and a pCR4-eGFP control with DpnI (20 U per reaction, 15–30 min at 37°C) to digest methylated template DNA. DpnI digestion of the mCherry PCR is optional if the template plasmid for mCherry CDS donor lacks an ampicillin resistance marker.

**Fig 2:**
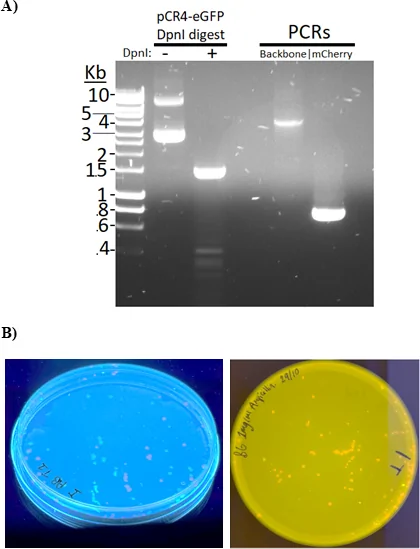
Representative results. (**A**) Agarose gel electrophoresis analysis of the undigested pCR4-eGFP (−) showing supercoiled and nicked vector, DpnI digested pCR4-eGFP vector (+) (multiple fragments) (left lanes), and PCR amplicons of pCR4 vector backbone (3,932 bp) and mCherry CDS insert (762 bp) (right lanes). (**B**) Plated transformation for pCR4 backbone and mCherry CDS PCR products. Colonies that result from successful recombination between the two PCR products fluoresce red when viewed under green light (left), while background colonies (likely resulting from transformation with pCR4-eGFP template that escaped DpnI digestion) fluoresce green under blue light (right; 4 o’clock position).

Students run 1% agarose gels to confirm PCR product sizes and DpnI activity. See the representative gel in Fig. 2.

While the agarose gel electrophoresis is underway, students prepare LB ampicillin agar plates and perform bacterial transformations with:

- No DNA (negative control).
- Undigested pCR4-eGFP (positive control; many green colonies expected).
- DpnI-digested pCR4-eGFP (should yield few/no colonies).
- DpnI-treated backbone PCR + mCherry PCR mix (expected red colonies upon successful IVA).

After transformation, students perform outgrowth recovery and then plate their transformation mixtures on LB-ampicillin and incubate overnight at 37°C.

### Session 3: colony observation (≈1 h, next day)

Count and score colonies:

- Successful IVA → red colonies. These are clearly distinguished by dark red coloration in daylight and bright red fluorescence under green light.
- Background → green colonies, from undigested template DNA escaping DpnI digestion. These have a pale, dim green coloration in daylight, but bright green fluorescence under blue light.

Success rate varies with pipetting precision and adherence to the digestion protocol; most groups obtain many red colonies with some green colonies, while some achieve a majority of red colonies and only one or two green colonies.

### Tips and tricks for successful implementation

- Homology arm design: 25 bp works reliably; longer arms (30–40 bp) can increase efficiency, though will raise primer cost.
- DpnI is critical: incomplete digestion is the most likely source of green background colonies. Always include the vector + DpnI control. Extend digestion time or enzyme amount if background persists.
- PCR optimization: Phusion gives clean products; verify annealing temperature if switching polymerases. Taq polymerase should be avoided as it introduces A overhangs to PCR products, which would disrupt the intended reading frame during insertion of the CDS into the vector.
- Competent cells: in-house CCMB80-prepared cells are economical and sufficiently competent. These can be prepared in advance of the practical class and stored at −80°C. On the day of Session 2, these can be stored on dry ice before they are provided to students for thawing on ice. Avoid repeated freeze-thaws.
- Simplified transformation recovery: the volume of SOC or LB media added post heat shock for recovery can be reduced to 100 μL. The entire final 200 μL volume can be plated, avoiding the need to centrifuge before plating. The outgrowth time can also be reduced to 30 min if necessary, without significantly affecting transformation efficiency.
- Visual engagement: the red/green switch engages students. Have students photograph plates under the different lighting for their reports/discussions.
- Troubleshooting few red colonies: check PCR product yields on a gel; ensure correct ratio of vector to insert (roughly equimolar); verify homology sequences.
- Scaling: for larger classes, rotate tasks within groups. For individual work, prepare more competent cells.
- Extensions: discuss the RAIR mechanism, compare IVA to Gibson/restriction cloning, or prepare and sequence successful plasmids.

### Representative results

All student groups (*n* = 10 groups of 4) obtained red fluorescent colonies, confirming IVA success. Efficiency varied; some groups had many colonies, mostly red, whereas others had few colonies in total or many colonies but an equal mixture of red and green. This variation is likely due primarily to pipetting issues or adherence to the protocol during DpnI digestion.

DpnI-treated vector controls yielded near-zero colonies, whereas undigested controls yielded abundant green colonies. Gel analysis consistently showed correct amplicon sizes and effective template digestion (Fig. 2).

This visual readout enables immediate discussion of concepts such as template carryover, recombination efficiency, and experimental controls.

## CONCLUSION

This IVA-based practical class modernizes cloning education by replacing tedious traditional restriction/ligation-based methods with a fast, reliable, visually rewarding workflow. Students gain hands-on experience with PCR, restriction digestion for template removal, bacterial transformation, and seamless assembly, mirroring contemporary research practices, while avoiding common pitfalls that demotivate novices. The practical class also provides a springboard to teach primer design, including the use of homology, though this requires an additional bioinformatics-focused session.

The class is low-cost, uses standard laboratory bacterial strains/equipment, and fits within tight timetables. Student feedback highlights the excitement of seeing their own red colonies as a strong motivator. It effectively teaches core principles and builds confidence in molecular techniques.

## SUPPLEMENTAL MATERIAL

Supplemental material jmbe.00089-26-s0001.docx Construction of pCR4-eGFP plasmids, primer sequences, plasmid maps and sequences, streamlined heat-shock transformation protocol, approximate reagent costs, and student handbook.

ASM does not own the copyrights to Supplemental Material that may be linked to, or accessed through, an article. The authors have granted ASM a non-exclusive, world-wide license to publish the Supplemental Material files. Please contact the corresponding author directly for reuse.
