# Supplementary material for: In vivo assembly to switch fluorescent protein expression in Escherichia coli: a simple laboratory class to introduce molecular cloning
Source: J Microbiol Biol Educ. 2026 Jul 15;27(2):e00089-26. doi: 10.1128/jmbe.00089-26 (PMC13520670; doi:10.1128/jmbe.00089-26)
Supplement: Supplementary file 1 [file jmbe.00089-26-s0001.docx]

**Supplementary material**

**(1) Construction of pCR4-eGFP plasmids**

To perform the practical class, students will need a template plasmid containing an eGFP coding sequence (CDS) under a constitutive bacterial promoter, allowing constitutive expression of eGFP in transformed *E. coli*.

We have deposited such a plasmid, pCR4-eGFP, with Addgene: <https://www.addgene.org/258561/>

Alternatively, here is a detailed method on its construction: We constructed pCR4-eGFP, by PCR amplification (with Phusion polymerase; New England Biolabs) of the eGFP CDS from a donor plasmid (pX461, a gift from Feng Zhang, Addgene plasmid #48140: Practically any plasmid containing an eGFP CDS is a suitable template for this), using the eGFP primers shown in **Supplementary Table 1.** The resulting 733 bp amplicon was ligated into linearised topoisomerase-bound pCR4Blunt plasmid vector as supplied in the Zero Blunt™ TOPO™ PCR Cloning Kit (Invitrogen), according to the manufacturer’s protocol. Ligation products were transformed into chemically competent DH5α *E. coli,* and transformation mixtures plated on ampicillin LB plates. Note that the pCR4Blunt plasmid vector contains both ampicillin and kanamycin resistance genes. Colonies containing assembled pCR4-eGFP, with correct orientation of the eGFP CDS, were easily distinguished by their bright green fluorescence under blue light, owing to eGFP expression. A successfully transformed colony was picked, inoculated into liquid in LB ampicillin, and the resulting culture midi-prepped. This midi-prepped plasmid was sequenced (Source Bioscience) with the M13R primer, confirming the correct sequence of the insert. **N.B.** The plasmid supplied to students must be generated in a Dam-positive *E. coli* strain (such as DH5α) so that it is sensitive to DpnI digestion.

**(2) Primer Sequences.**

**Supplementary Table 1.** Sequences of primers used in the construction of the template plasmid, pCR4-eGFP, and for sequence confirmation.

| **Primer name** | **Sequence (5’- -3’)** |
| --- | --- |
| eGFP insert Forward | CTCGAGGTGAGCAAGGGCGAGG |
| eGFP insert Reverse | GGATCCTTCATTACTTGTACAGCTCGTCC |
| M13R | CAGGAAACAGCTATGACC |

**(3) Plasmid maps and sequences.**

>pCR4-eGFP

AACGCCAGCAACGCGGCCTTTTTACGGTTCCTGGCCTTTTGCTGGCCTTTTGCTCACATGTTCTTTCCTGCGTTATCCCCTGATTCTGTGGATAACCGTATTACCGCCTTTGAGTGAGCTGATACCGCTCGCCGCAGCCGAACGACCGAGCGCAGCGAGTCAGTGAGCGAGGAAGCGGAAGAGCGCCCAATACGCAAACCGCCTCTCCCCGCGCGTTGGCCGATTCATTAATGCAGCTGGCACGACAGGTTTCCCGACTGGAAAGCGGGCAGTGAGCGCAACGCAATTAATGTGAGTTAGCTCACTCATTAGGCACCCCAGGCTTTACACTTTATGCTTCCGGCTCGTATGTTGTGTGGAATTGTGAGCGGATAACAATTTCACACAGGAAACAGCTATGACCATGATTACGCCAAGCTCAGAATTAACCCTCACTAAAGGGACTAGTCCTGCAGGTTTAAACGAATTCGCCCTTCTCGAGGTGAGCAAGGGCGAGGAGCTGTTCACCGGGGTGGTGCCCATCCTGGTCGAGCTGGACGGCGACGTAAACGGCCACAAGTTCAGCGTGTCCGGCGAGGGCGAGGGCGATGCCACCTACGGCAAGCTGACCCTGAAGTTCATCTGCACCACCGGCAAGCTGCCCGTGCCCTGGCCCACCCTCGTGACCACCCTGACCTACGGCGTGCAGTGCTTCAGCCGCTACCCCGACCACATGAAGCAGCACGACTTCTTCAAGTCCGCCATGCCCGAAGGCTACGTCCAGGAGCGCACCATCTTCTTCAAGGACGACGGCAACTACAAGACCCGCGCCGAGGTGAAGTTCGAGGGCGACACCCTGGTGAACCGCATCGAGCTGAAGGGCATCGACTTCAAGGAGGACGGCAACATCCTGGGGCACAAGCTGGAGTACAACTACAACAGCCACAACGTCTATATCATGGCCGACAAGCAGAAGAACGGCATCAAGGTGAACTTCAAGATCCGCCACAACATCGAGGACGGCAGCGTGCAGCTCGCCGACCACTACCAGCAGAACACCCCCATCGGCGACGGCCCCGTGCTGCTGCCCGACAACCACTACCTGAGCACCCAGTCCGCCCTGAGCAAAGACCCCAACGAGAAGCGCGATCACATGGTCCTGCTGGAGTTCGTGACCGCCGCCGGGATCACTCTCGGCATGGACGAGCTGTACAAGTAATGAAGGATCCAAGGGCGAATTCGCGGCCGCTAAATTCAATTCGCCCTATAGTGAGTCGTATTACAATTCACTGGCCGTCGTTTTACAACGTCGTGACTGGGAAAACCCTGGCGTTACCCAACTTAATCGCCTTGCAGCACATCCCCCTTTCGCCAGCTGGCGTAATAGCGAAGAGGCCCGCACCGATCGCCCTTCCCAACAGTTGCGCAGCCTATACGTACGGCAGTTTAAGGTTTACACCTATAAAAGAGAGAGCCGTTATCGTCTGTTTGTGGATGTACAGAGTGATATTATTGACACGCCGGGGCGACGGATGGTGATCCCCCTGGCCAGTGCACGTCTGCTGTCAGATAAAGTCTCCCGTGAACTTTACCCGGTGGTGCATATCGGGGATGAAAGCTGGCGCATGATGACCACCGATATGGCCAGTGTGCCGGTCTCCGTTATCGGGGAAGAAGTGGCTGATCTCAGCCACCGCGAAAATGACATCAAAAACGCCATTAACCTGATGTTCTGGGGAATATAAATGTCAGGCATGAGATTATCAAAAAGGATCTTCACCTAGATCCTTTTCACGTAGAAAGCCAGTCCGCAGAAACGGTGCTGACCCCGGATGAATGTCAGCTACTGGGCTATCTGGACAAGGGAAAACGCAAGCGCAAAGAGAAAGCAGGTAGCTTGCAGTGGGCTTACATGGCGATAGCTAGACTGGGCGGTTTTATGGACAGCAAGCGAACCGGAATTGCCAGCTGGGGCGCCCTCTGGTAAGGTTGGGAAGCCCTGCAAAGTAAACTGGATGGCTTTCTTGCCGCCAAGGATCTGATGGCGCAGGGGATCAAGCTCTGATCAAGAGACAGGATGAGGATCGTTTCGCATGATTGAACAAGATGGATTGCACGCAGGTTCTCCGGCCGCTTGGGTGGAGAGGCTATTCGGCTATGACTGGGCACAACAGACAATCGGCTGCTCTGATGCCGCCGTGTTCCGGCTGTCAGCGCAGGGGCGCCCGGTTCTTTTTGTCAAGACCGACCTGTCCGGTGCCCTGAATGAACTGCAAGACGAGGCAGCGCGGCTATCGTGGCTGGCCACGACGGGCGTTCCTTGCGCAGCTGTGCTCGACGTTGTCACTGAAGCGGGAAGGGACTGGCTGCTATTGGGCGAAGTGCCGGGGCAGGATCTCCTGTCATCTCACCTTGCTCCTGCCGAGAAAGTATCCATCATGGCTGATGCAATGCGGCGGCTGCATACGCTTGATCCGGCTACCTGCCCATTCGACCACCAAGCGAAACATCGCATCGAGCGAGCACGTACTCGGATGGAAGCCGGTCTTGTCGATCAGGATGATCTGGACGAAGAGCATCAGGGGCTCGCGCCAGCCGAACTGTTCGCCAGGCTCAAGGCGAGCATGCCCGACGGCGAGGATCTCGTCGTGACCCATGGCGATGCCTGCTTGCCGAATATCATGGTGGAAAATGGCCGCTTTTCTGGATTCATCGACTGTGGCCGGCTGGGTGTGGCGGACCGCTATCAGGACATAGCGTTGGCTACCCGTGATATTGCTGAAGAGCTTGGCGGCGAATGGGCTGACCGCTTCCTCGTGCTTTACGGTATCGCCGCTCCCGATTCGCAGCGCATCGCCTTCTATCGCCTTCTTGACGAGTTCTTCTGAATTATTAACGCTTACAATTTCCTGATGCGGTATTTTCTCCTTACGCATCTGTGCGGTATTTCACACCGCATCAGGTGGCACTTTTCGGGGAAATGTGCGCGGAACCCCTATTTGTTTATTTTTCTAAATACATTCAAATATGTATCCGCTCATGAGATTATCAAAAAGGATCTTCACCTAGATCCTTTTAAATTAAAAATGAAGTTTTAAATCAATCTAAAGTATATATGAGTAAACTTGGTCTGACAGTTACCAATGCTTAATCAGTGAGGCACCTATCTCAGCGATCTGTCTATTTCGTTCATCCATAGTTGCCTGACTCCCCGTCGTGTAGATAACTACGATACGGGAGGGCTTACCATCTGGCCCCAGTGCTGCAATGATACCGCGAGACCCACGCTCACCGGCTCCAGATTTATCAGCAATAAACCAGCCAGCCGGAAGGGCCGAGCGCAGAAGTGGTCCTGCAACTTTATCCGCCTCCATCCAGTCTATTAATTGTTGCCGGGAAGCTAGAGTAAGTAGTTCGCCAGTTAATAGTTTGCGCAACGTTGTTGCCATTGCTACAGGCATCGTGGTGTCACGCTCGTCGTTTGGTATGGCTTCATTCAGCTCCGGTTCCCAACGATCAAGGCGAGTTACATGATCCCCCATGTTGTGCAAAAAAGCGGTTAGCTCCTTCGGTCCTCCGATCGTTGTCAGAAGTAAGTTGGCCGCAGTGTTATCACTCATGGTTATGGCAGCACTGCATAATTCTCTTACTGTCATGCCATCCGTAAGATGCTTTTCTGTGACTGGTGAGTACTCAACCAAGTCATTCTGAGAATAGTGTATGCGGCGACCGAGTTGCTCTTGCCCGGCGTCAATACGGGATAATACCGCGCCACATAGCAGAACTTTAAAAGTGCTCATCATTGGAAAACGTTCTTCGGGGCGAAAACTCTCAAGGATCTTACCGCTGTTGAGATCCAGTTCGATGTAACCCACTCGTGCACCCAACTGATCTTCAGCATCTTTTACTTTCACCAGCGTTTCTGGGTGAGCAAAAACAGGAAGGCAAAATGCCGCAAAAAAGGGAATAAGGGCGACACGGAAATGTTGAATACTCATACTCTTCCTTTTTCAATATTATTGAAGCATTTATCAGGGTTATTGTCTCATGACCAAAATCCCTTAACGTGAGTTTTCGTTCCACTGAGCGTCAGACCCCGTAGAAAAGATCAAAGGATCTTCTTGAGATCCTTTTTTTCTGCGCGTAATCTGCTGCTTGCAAACAAAAAAACCACCGCTACCAGCGGTGGTTTGTTTGCCGGATCAAGAGCTACCAACTCTTTTTCCGAAGGTAACTGGCTTCAGCAGAGCGCAGATACCAAATACTGTTCTTCTAGTGTAGCCGTAGTTAGGCCACCACTTCAAGAACTCTGTAGCACCGCCTACATACCTCGCTCTGCTAATCCTGTTACCAGTGGCTGCTGCCAGTGGCGATAAGTCGTGTCTTACCGGGTTGGACTCAAGACGATAGTTACCGGATAAGGCGCAGCGGTCGGGCTGAACGGGGGGTTCGTGCACACAGCCCAGCTTGGAGCGAACGACCTACACCGAACTGAGATACCTACAGCGTGAGCTATGAGAAAGCGCCACGCTTCCCGAAGGGAGAAAGGCGGACAGGTATCCGGTAAGCGGCAGGGTCGGAACAGGAGAGCGCACGAGGGAGCTTCCAGGGGGAAACGCCTGGTATCTTTATAGTCCTGTCGGGTTTCGCCACCTCTGACTTGAGCGTCGATTTTTGTGATGCTCGTCAGGGGGGCGGAGCCTATGGAAA

>pCR4-eGFP_map

>pCR4-mCherry

AACGCCAGCAACGCGGCCTTTTTACGGTTCCTGGCCTTTTGCTGGCCTTTTGCTCACATGTTCTTTCCTGCGTTATCCCCTGATTCTGTGGATAACCGTATTACCGCCTTTGAGTGAGCTGATACCGCTCGCCGCAGCCGAACGACCGAGCGCAGCGAGTCAGTGAGCGAGGAAGCGGAAGAGCGCCCAATACGCAAACCGCCTCTCCCCGCGCGTTGGCCGATTCATTAATGCAGCTGGCACGACAGGTTTCCCGACTGGAAAGCGGGCAGTGAGCGCAACGCAATTAATGTGAGTTAGCTCACTCATTAGGCACCCCAGGCTTTACACTTTATGCTTCCGGCTCGTATGTTGTGTGGAATTGTGAGCGGATAACAATTTCACACAGGAAACAGCTATGACCATGATTACGCCAAGCTCAGAATTAACCCTCACTAAAGGGACTAGTCCTGCAGGTTTAAACGTGAGCAAGGGCGAGGAGGATAACATGGCCATCATCAAGGAGTTCATGCGCTTCAAGGTGCACATGGAGGGCTCCGTGAACGGCCACGAGTTCGAGATCGAGGGCGAGGGCGAGGGCCGCCCCTACGAGGGCACCCAGACCGCCAAGCTGAAGGTGACCAAGGGTGGCCCCCTGCCCTTCGCCTGGGACATCCTGTCCCCTCAGTTCATGTACGGCTCCAAGGCCTACGTGAAGCACCCCGCCGACATCCCCGACTACTTGAAGCTGTCCTTCCCCGAGGGCTTCAAGTGGGAGCGCGTGATGAACTTCGAGGACGGCGGCGTGGTGACCGTGACCCAGGACTCCTCCCTGCAGGACGGCGAGTTCATCTACAAGGTGAAGCTGCGCGGCACCAACTTCCCCTCCGACGGCCCCGTAATGCAGAAGAAGACCATGGGCTGGGAGGCCTCCTCCGAGCGGATGTACCCCGAGGACGGCGCCCTGAAGGGCGAGATCAAGCAGAGGCTGAAGCTGAAGGACGGCGGCCACTACGACGCTGAGGTCAAGACCACCTACAAGGCCAAGAAGCCCGTGCAGCTGCCCGGCGCCTACAACGTCAACATCAAGTTGGACATCACCTCCCACAACGAGGACTACACCATCGTGGAACAGTACGAACGCGCCGAGGGCCGCCACTCCACCGGCGGCATGGACGAGCTGTACAAGTAATGAACGGCCGCTAAATTCAATTCGCCCTATAGTGAGTCGTATTACAATTCACTGGCCGTCGTTTTACAACGTCGTGACTGGGAAAACCCTGGCGTTACCCAACTTAATCGCCTTGCAGCACATCCCCCTTTCGCCAGCTGGCGTAATAGCGAAGAGGCCCGCACCGATCGCCCTTCCCAACAGTTGCGCAGCCTATACGTACGGCAGTTTAAGGTTTACACCTATAAAAGAGAGAGCCGTTATCGTCTGTTTGTGGATGTACAGAGTGATATTATTGACACGCCGGGGCGACGGATGGTGATCCCCCTGGCCAGTGCACGTCTGCTGTCAGATAAAGTCTCCCGTGAACTTTACCCGGTGGTGCATATCGGGGATGAAAGCTGGCGCATGATGACCACCGATATGGCCAGTGTGCCGGTCTCCGTTATCGGGGAAGAAGTGGCTGATCTCAGCCACCGCGAAAATGACATCAAAAACGCCATTAACCTGATGTTCTGGGGAATATAAATGTCAGGCATGAGATTATCAAAAAGGATCTTCACCTAGATCCTTTTCACGTAGAAAGCCAGTCCGCAGAAACGGTGCTGACCCCGGATGAATGTCAGCTACTGGGCTATCTGGACAAGGGAAAACGCAAGCGCAAAGAGAAAGCAGGTAGCTTGCAGTGGGCTTACATGGCGATAGCTAGACTGGGCGGTTTTATGGACAGCAAGCGAACCGGAATTGCCAGCTGGGGCGCCCTCTGGTAAGGTTGGGAAGCCCTGCAAAGTAAACTGGATGGCTTTCTTGCCGCCAAGGATCTGATGGCGCAGGGGATCAAGCTCTGATCAAGAGACAGGATGAGGATCGTTTCGCATGATTGAACAAGATGGATTGCACGCAGGTTCTCCGGCCGCTTGGGTGGAGAGGCTATTCGGCTATGACTGGGCACAACAGACAATCGGCTGCTCTGATGCCGCCGTGTTCCGGCTGTCAGCGCAGGGGCGCCCGGTTCTTTTTGTCAAGACCGACCTGTCCGGTGCCCTGAATGAACTGCAAGACGAGGCAGCGCGGCTATCGTGGCTGGCCACGACGGGCGTTCCTTGCGCAGCTGTGCTCGACGTTGTCACTGAAGCGGGAAGGGACTGGCTGCTATTGGGCGAAGTGCCGGGGCAGGATCTCCTGTCATCTCACCTTGCTCCTGCCGAGAAAGTATCCATCATGGCTGATGCAATGCGGCGGCTGCATACGCTTGATCCGGCTACCTGCCCATTCGACCACCAAGCGAAACATCGCATCGAGCGAGCACGTACTCGGATGGAAGCCGGTCTTGTCGATCAGGATGATCTGGACGAAGAGCATCAGGGGCTCGCGCCAGCCGAACTGTTCGCCAGGCTCAAGGCGAGCATGCCCGACGGCGAGGATCTCGTCGTGACCCATGGCGATGCCTGCTTGCCGAATATCATGGTGGAAAATGGCCGCTTTTCTGGATTCATCGACTGTGGCCGGCTGGGTGTGGCGGACCGCTATCAGGACATAGCGTTGGCTACCCGTGATATTGCTGAAGAGCTTGGCGGCGAATGGGCTGACCGCTTCCTCGTGCTTTACGGTATCGCCGCTCCCGATTCGCAGCGCATCGCCTTCTATCGCCTTCTTGACGAGTTCTTCTGAATTATTAACGCTTACAATTTCCTGATGCGGTATTTTCTCCTTACGCATCTGTGCGGTATTTCACACCGCATCAGGTGGCACTTTTCGGGGAAATGTGCGCGGAACCCCTATTTGTTTATTTTTCTAAATACATTCAAATATGTATCCGCTCATGAGATTATCAAAAAGGATCTTCACCTAGATCCTTTTAAATTAAAAATGAAGTTTTAAATCAATCTAAAGTATATATGAGTAAACTTGGTCTGACAGTTACCAATGCTTAATCAGTGAGGCACCTATCTCAGCGATCTGTCTATTTCGTTCATCCATAGTTGCCTGACTCCCCGTCGTGTAGATAACTACGATACGGGAGGGCTTACCATCTGGCCCCAGTGCTGCAATGATACCGCGAGACCCACGCTCACCGGCTCCAGATTTATCAGCAATAAACCAGCCAGCCGGAAGGGCCGAGCGCAGAAGTGGTCCTGCAACTTTATCCGCCTCCATCCAGTCTATTAATTGTTGCCGGGAAGCTAGAGTAAGTAGTTCGCCAGTTAATAGTTTGCGCAACGTTGTTGCCATTGCTACAGGCATCGTGGTGTCACGCTCGTCGTTTGGTATGGCTTCATTCAGCTCCGGTTCCCAACGATCAAGGCGAGTTACATGATCCCCCATGTTGTGCAAAAAAGCGGTTAGCTCCTTCGGTCCTCCGATCGTTGTCAGAAGTAAGTTGGCCGCAGTGTTATCACTCATGGTTATGGCAGCACTGCATAATTCTCTTACTGTCATGCCATCCGTAAGATGCTTTTCTGTGACTGGTGAGTACTCAACCAAGTCATTCTGAGAATAGTGTATGCGGCGACCGAGTTGCTCTTGCCCGGCGTCAATACGGGATAATACCGCGCCACATAGCAGAACTTTAAAAGTGCTCATCATTGGAAAACGTTCTTCGGGGCGAAAACTCTCAAGGATCTTACCGCTGTTGAGATCCAGTTCGATGTAACCCACTCGTGCACCCAACTGATCTTCAGCATCTTTTACTTTCACCAGCGTTTCTGGGTGAGCAAAAACAGGAAGGCAAAATGCCGCAAAAAAGGGAATAAGGGCGACACGGAAATGTTGAATACTCATACTCTTCCTTTTTCAATATTATTGAAGCATTTATCAGGGTTATTGTCTCATGACCAAAATCCCTTAACGTGAGTTTTCGTTCCACTGAGCGTCAGACCCCGTAGAAAAGATCAAAGGATCTTCTTGAGATCCTTTTTTTCTGCGCGTAATCTGCTGCTTGCAAACAAAAAAACCACCGCTACCAGCGGTGGTTTGTTTGCCGGATCAAGAGCTACCAACTCTTTTTCCGAAGGTAACTGGCTTCAGCAGAGCGCAGATACCAAATACTGTTCTTCTAGTGTAGCCGTAGTTAGGCCACCACTTCAAGAACTCTGTAGCACCGCCTACATACCTCGCTCTGCTAATCCTGTTACCAGTGGCTGCTGCCAGTGGCGATAAGTCGTGTCTTACCGGGTTGGACTCAAGACGATAGTTACCGGATAAGGCGCAGCGGTCGGGCTGAACGGGGGGTTCGTGCACACAGCCCAGCTTGGAGCGAACGACCTACACCGAACTGAGATACCTACAGCGTGAGCTATGAGAAAGCGCCACGCTTCCCGAAGGGAGAAAGGCGGACAGGTATCCGGTAAGCGGCAGGGTCGGAACAGGAGAGCGCACGAGGGAGCTTCCAGGGGGAAACGCCTGGTATCTTTATAGTCCTGTCGGGTTTCGCCACCTCTGACTTGAGCGTCGATTTTTGTGATGCTCGTCAGGGGGGCGGAGCCTATGGAAA

>pCR4-mCherry_map

>pCR4_backbone_PCR_product

CGGCCGCTAAATTCAATTCGCCCTATAGTGAGTCGTATTACAATTCACTGGCCGTCGTTTTACAACGTCGTGACTGGGAAAACCCTGGCGTTACCCAACTTAATCGCCTTGCAGCACATCCCCCTTTCGCCAGCTGGCGTAATAGCGAAGAGGCCCGCACCGATCGCCCTTCCCAACAGTTGCGCAGCCTATACGTACGGCAGTTTAAGGTTTACACCTATAAAAGAGAGAGCCGTTATCGTCTGTTTGTGGATGTACAGAGTGATATTATTGACACGCCGGGGCGACGGATGGTGATCCCCCTGGCCAGTGCACGTCTGCTGTCAGATAAAGTCTCCCGTGAACTTTACCCGGTGGTGCATATCGGGGATGAAAGCTGGCGCATGATGACCACCGATATGGCCAGTGTGCCGGTCTCCGTTATCGGGGAAGAAGTGGCTGATCTCAGCCACCGCGAAAATGACATCAAAAACGCCATTAACCTGATGTTCTGGGGAATATAAATGTCAGGCATGAGATTATCAAAAAGGATCTTCACCTAGATCCTTTTCACGTAGAAAGCCAGTCCGCAGAAACGGTGCTGACCCCGGATGAATGTCAGCTACTGGGCTATCTGGACAAGGGAAAACGCAAGCGCAAAGAGAAAGCAGGTAGCTTGCAGTGGGCTTACATGGCGATAGCTAGACTGGGCGGTTTTATGGACAGCAAGCGAACCGGAATTGCCAGCTGGGGCGCCCTCTGGTAAGGTTGGGAAGCCCTGCAAAGTAAACTGGATGGCTTTCTTGCCGCCAAGGATCTGATGGCGCAGGGGATCAAGCTCTGATCAAGAGACAGGATGAGGATCGTTTCGCATGATTGAACAAGATGGATTGCACGCAGGTTCTCCGGCCGCTTGGGTGGAGAGGCTATTCGGCTATGACTGGGCACAACAGACAATCGGCTGCTCTGATGCCGCCGTGTTCCGGCTGTCAGCGCAGGGGCGCCCGGTTCTTTTTGTCAAGACCGACCTGTCCGGTGCCCTGAATGAACTGCAAGACGAGGCAGCGCGGCTATCGTGGCTGGCCACGACGGGCGTTCCTTGCGCAGCTGTGCTCGACGTTGTCACTGAAGCGGGAAGGGACTGGCTGCTATTGGGCGAAGTGCCGGGGCAGGATCTCCTGTCATCTCACCTTGCTCCTGCCGAGAAAGTATCCATCATGGCTGATGCAATGCGGCGGCTGCATACGCTTGATCCGGCTACCTGCCCATTCGACCACCAAGCGAAACATCGCATCGAGCGAGCACGTACTCGGATGGAAGCCGGTCTTGTCGATCAGGATGATCTGGACGAAGAGCATCAGGGGCTCGCGCCAGCCGAACTGTTCGCCAGGCTCAAGGCGAGCATGCCCGACGGCGAGGATCTCGTCGTGACCCATGGCGATGCCTGCTTGCCGAATATCATGGTGGAAAATGGCCGCTTTTCTGGATTCATCGACTGTGGCCGGCTGGGTGTGGCGGACCGCTATCAGGACATAGCGTTGGCTACCCGTGATATTGCTGAAGAGCTTGGCGGCGAATGGGCTGACCGCTTCCTCGTGCTTTACGGTATCGCCGCTCCCGATTCGCAGCGCATCGCCTTCTATCGCCTTCTTGACGAGTTCTTCTGAATTATTAACGCTTACAATTTCCTGATGCGGTATTTTCTCCTTACGCATCTGTGCGGTATTTCACACCGCATCAGGTGGCACTTTTCGGGGAAATGTGCGCGGAACCCCTATTTGTTTATTTTTCTAAATACATTCAAATATGTATCCGCTCATGAGATTATCAAAAAGGATCTTCACCTAGATCCTTTTAAATTAAAAATGAAGTTTTAAATCAATCTAAAGTATATATGAGTAAACTTGGTCTGACAGTTACCAATGCTTAATCAGTGAGGCACCTATCTCAGCGATCTGTCTATTTCGTTCATCCATAGTTGCCTGACTCCCCGTCGTGTAGATAACTACGATACGGGAGGGCTTACCATCTGGCCCCAGTGCTGCAATGATACCGCGAGACCCACGCTCACCGGCTCCAGATTTATCAGCAATAAACCAGCCAGCCGGAAGGGCCGAGCGCAGAAGTGGTCCTGCAACTTTATCCGCCTCCATCCAGTCTATTAATTGTTGCCGGGAAGCTAGAGTAAGTAGTTCGCCAGTTAATAGTTTGCGCAACGTTGTTGCCATTGCTACAGGCATCGTGGTGTCACGCTCGTCGTTTGGTATGGCTTCATTCAGCTCCGGTTCCCAACGATCAAGGCGAGTTACATGATCCCCCATGTTGTGCAAAAAAGCGGTTAGCTCCTTCGGTCCTCCGATCGTTGTCAGAAGTAAGTTGGCCGCAGTGTTATCACTCATGGTTATGGCAGCACTGCATAATTCTCTTACTGTCATGCCATCCGTAAGATGCTTTTCTGTGACTGGTGAGTACTCAACCAAGTCATTCTGAGAATAGTGTATGCGGCGACCGAGTTGCTCTTGCCCGGCGTCAATACGGGATAATACCGCGCCACATAGCAGAACTTTAAAAGTGCTCATCATTGGAAAACGTTCTTCGGGGCGAAAACTCTCAAGGATCTTACCGCTGTTGAGATCCAGTTCGATGTAACCCACTCGTGCACCCAACTGATCTTCAGCATCTTTTACTTTCACCAGCGTTTCTGGGTGAGCAAAAACAGGAAGGCAAAATGCCGCAAAAAAGGGAATAAGGGCGACACGGAAATGTTGAATACTCATACTCTTCCTTTTTCAATATTATTGAAGCATTTATCAGGGTTATTGTCTCATGACCAAAATCCCTTAACGTGAGTTTTCGTTCCACTGAGCGTCAGACCCCGTAGAAAAGATCAAAGGATCTTCTTGAGATCCTTTTTTTCTGCGCGTAATCTGCTGCTTGCAAACAAAAAAACCACCGCTACCAGCGGTGGTTTGTTTGCCGGATCAAGAGCTACCAACTCTTTTTCCGAAGGTAACTGGCTTCAGCAGAGCGCAGATACCAAATACTGTTCTTCTAGTGTAGCCGTAGTTAGGCCACCACTTCAAGAACTCTGTAGCACCGCCTACATACCTCGCTCTGCTAATCCTGTTACCAGTGGCTGCTGCCAGTGGCGATAAGTCGTGTCTTACCGGGTTGGACTCAAGACGATAGTTACCGGATAAGGCGCAGCGGTCGGGCTGAACGGGGGGTTCGTGCACACAGCCCAGCTTGGAGCGAACGACCTACACCGAACTGAGATACCTACAGCGTGAGCTATGAGAAAGCGCCACGCTTCCCGAAGGGAGAAAGGCGGACAGGTATCCGGTAAGCGGCAGGGTCGGAACAGGAGAGCGCACGAGGGAGCTTCCAGGGGGAAACGCCTGGTATCTTTATAGTCCTGTCGGGTTTCGCCACCTCTGACTTGAGCGTCGATTTTTGTGATGCTCGTCAGGGGGGCGGAGCCTATGGAAAAACGCCAGCAACGCGGCCTTTTTACGGTTCCTGGCCTTTTGCTGGCCTTTTGCTCACATGTTCTTTCCTGCGTTATCCCCTGATTCTGTGGATAACCGTATTACCGCCTTTGAGTGAGCTGATACCGCTCGCCGCAGCCGAACGACCGAGCGCAGCGAGTCAGTGAGCGAGGAAGCGGAAGAGCGCCCAATACGCAAACCGCCTCTCCCCGCGCGTTGGCCGATTCATTAATGCAGCTGGCACGACAGGTTTCCCGACTGGAAAGCGGGCAGTGAGCGCAACGCAATTAATGTGAGTTAGCTCACTCATTAGGCACCCCAGGCTTTACACTTTATGCTTCCGGCTCGTATGTTGTGTGGAATTGTGAGCGGATAACAATTTCACACAGGAAACAGCTATGACCATGATTACGCCAAGCTCAGAATTAACCCTCACTAAAGGGACTAGTCCTGCAGGTTTAAAC

>pCR4_backbone_PCR_product_map

>mCherry_PCR_product

AGGGACTAGTCCTGCAGGTTTAAACGTGAGCAAGGGCGAGGAGGATAACATGGCCATCATCAAGGAGTTCATGCGCTTCAAGGTGCACATGGAGGGCTCCGTGAACGGCCACGAGTTCGAGATCGAGGGCGAGGGCGAGGGCCGCCCCTACGAGGGCACCCAGACCGCCAAGCTGAAGGTGACCAAGGGTGGCCCCCTGCCCTTCGCCTGGGACATCCTGTCCCCTCAGTTCATGTACGGCTCCAAGGCCTACGTGAAGCACCCCGCCGACATCCCCGACTACTTGAAGCTGTCCTTCCCCGAGGGCTTCAAGTGGGAGCGCGTGATGAACTTCGAGGACGGCGGCGTGGTGACCGTGACCCAGGACTCCTCCCTGCAGGACGGCGAGTTCATCTACAAGGTGAAGCTGCGCGGCACCAACTTCCCCTCCGACGGCCCCGTAATGCAGAAGAAGACCATGGGCTGGGAGGCCTCCTCCGAGCGGATGTACCCCGAGGACGGCGCCCTGAAGGGCGAGATCAAGCAGAGGCTGAAGCTGAAGGACGGCGGCCACTACGACGCTGAGGTCAAGACCACCTACAAGGCCAAGAAGCCCGTGCAGCTGCCCGGCGCCTACAACGTCAACATCAAGTTGGACATCACCTCCCACAACGAGGACTACACCATCGTGGAACAGTACGAACGCGCCGAGGGCCGCCACTCCACCGGCGGCATGGACGAGCTGTACAAGTAATGAACGGCCGCTAAATTCAATTCGCCCTA

>mCherry_PCR_product_map

**(4) Streamlined heat-shock transformation protocol**

Heat-shock transformation is, of course, a foundational method in microbiology. It is amenable to small modifications to simplify the procedure and reduce the total time taken. The trade-off is that these modifications slightly reduce the final transformation efficiency, but since it proved sufficient for the students to attain good colony numbers, we feel this is a worthwhile trade-off for a busy practical class.

Student groups were given one 100 μL aliquot of competent cells per transformation. These were prepared in-house by the CCMB80 method [10] ahead of the class, stored at -80 ⁰C until the day of the class, then held on dry ice until given to the students.

Here is the streamlined heat-shock transformation method that we used in the practical class:

Student groups:

- Receive their competent cell aliquots and thaw on wet ice for 10 minutes.
- Meanwhile, they label four 1.5 mL micro-centrifuge tubes, place them on ice, and add the corresponding transforming DNA:
  - 5 μL DpnI-treated vector PCR product & 5 μl DpnI-treated insert PCR product. Mix these by pipetting.
  - 10 μL distilled H_2_O (a negative control).
  - 10 μL 500 ng/μL pCR4-EGFP, treated with DpnI (control reaction for DpnI activity, should yield no colonies).
  - 10 μL 500 ng/μL pCR4-EGFP, NOT treated with DpnI (positive control for transformation and eGFP expression).
- Add 100 μL of thawed competent cells to each of these and incubate on ice for 30 minutes.
- Perform heat shock - Place tubes at 42 ⁰C for 2 minutes. (Ideally, this is performed with a water bath. We were limited to a dry microtube heat block. It still worked).
- Immediately place on ice and incubate on ice for 5 minutes.
- Add 100 μL LB (ideally Super Optimal Broth (SOC) would be used instead of LB).
- Place transformation tubes in a suitably sized container and place inside a shaking incubator at 37 ⁰C and 200 RPM. Incubate for 30 minutes (since the selecting antibiotic is ampicillin, the length of this recovery step has been reduced to 30 minutes).
- Gently mix to resuspend cells, then plate the full volume (~200 μL) of each transformation reaction on LB-agar ampicillin (100 μg/mL) plates.
- Incubate overnight at 37 ⁰

**(5) Approximate reagent costs**

**PCR reagents**

Primers - The listed primers were purchased from Merck at 25 nmol scale for 10p per base (total cost approximately $20).

Enzymes - Phusion polymerase kit (100 units) and DpnI (1000 units) were purchased from NEB. One pack of each was just sufficient for the practical class of 40 students. Total cost of enzymes was approximately $200.

**Competent cells**

We purchased sufficient quantities of the various salt components of CCMB80 buffer that we could have made at least 20 L of buffer, a volume sufficient to make competent cells for more than 40 practical classes of the size we ran (40 students). The final cost of these components was around $200. Alternatively, numerous suppliers stock 500 ml bottles of pre-made CCMB80 buffer for prices around $50. The calcium chloride method would be cheaper, but since the transformation efficiency with this method is generally lower than the CCMB80 method, we used the latter to give students a higher chance of success. In-house production is much less expensive than purchasing the required quantity of competent cells from commercial suppliers.

**(6) Student handbook.**

**N.B. for preparation of the laboratory class: This handbook was written with concentrations of several of the PCR reagent and DpnI stocks adjusted for ease for students with limited pipetting experience.**

**Aims and Learning Outcomes**

**Summary**

Molecular cloning is a group of methods used to isolate DNA sequences and incorporate them into vectors such as plasmids or artificial chromosomes, which can then be propagated in host organisms. In this class you will perform *in vivo* assembly cloning.

**Aims**

To introduce you to several techniques commonly used in molecular biology, and to help you develop the practical skills needed to use these techniques.

**Learning Objectives**

(a) Skills. On successful completion of this practical class students should be able to:

•	carry out accurate pipetting for PCR

•	pour LB plates and agarose gels

•	perform a bacterial transformation

(b) Knowledge and understanding. On completion of this practical class students should have gained an understanding of the principles underlying the procedures listed above and the concepts explained in the handbook.

**Preparation**

Read this protocol before the practical class.

**Protocol**

**Day 1**

**1. DNA preparation**

Label TWO 200μL PCR tubes with your initials and numbers 1 and 2.

**Tube 1 - pCR4-EGFP**

Preparation of the vector pCR4-EGFP plasmid – pipette the volumes shown below into tube 1

| **Reagent (stock concentration)** | **Volume (μL)** |
| --- | --- |
| HF buffer (5x concentrated) | 5 |
| dNTP mix (1 mM) | 5 |
| Backbone primer mix (2.5 μM) | 5 |
| pCR4-EGFP (10 ng/μL) | 5 |
| Phusion polymerase (0.1 Units/μl) | 5 |

**Tube 2 - mCherry Fragment**

Preparation of the mCherry coding sequence insert - pipette the volumes shown below into tube 2.

| **Reagent (stock concentration)** | **Volume (μL)** |
| --- | --- |
| HF buffer (5x concentrated) | 5 |
| dNTP mix (1 mM) | 5 |
| mCherry primer mix (2.5 μM) | 5 |
| mCherry CDS (10 ng/μL) | 5 |
| Phusion polymerase (0.1 Units/μl) | 5 |

**2. Running the PCR**

Place labelled PCR tubes in PCR machine. Make sure your initials are on the tubes.

|  | Initial denaturation | PCR cycling  30 cycles | | | Final extension | Hold |
| --- | --- | --- | --- | --- | --- | --- |
| Temp  (⁰C) | 98 | 98 | 62 | 72 | 72 | 10 |
| Time (Min:Sec) | 3:00 | 0:15 | 0:15 | 2:00 | 5:00 | 2:00 |

**3. DpnI digestion of the starting template**

You will conduct a test for DpnI activity on some original template DNA.

Label two PCR tubes **3** and **4** and add your initials. Then prepare the following reactions in these tubes and keep them on ice.

| **Reagent** | **Tube 3: DpnI +’ve reaction**  **Volume (μl)**  **(digested)** | **Tube 4: DpnI -’ve reaction**  **Volume (μl)**  **(undigested)** |
| --- | --- | --- |
| **5x HF buffer** | **5** | **5** |
| **1 μg/μl pCR4-eGFP** | **1** | **1** |
| **DpnI (4 units/μL)** | **5** | **0** |
| **ddH_2_O** | **14** | **19** |

**4. Pour LB agar plates**

While the PCR is running you can pour LB agar plates containing ampicillin (the selection antibiotic).

(stock is 1000mg/ml)- add 100μl of this stock to 100ml of hand hot LB agar

The final concentration will be 1mg/ml ampicillin

**See the demonstration first**

-all groups will pour plates – each person in the group will pour one plate.

**Demonstrators will help with the gel and agar plate pouring**

**Take off gloves before handling a Bunsen burner flame**

You can take a break here and return to coincide with end of the PCR.

**5. Digestion of the PCR products**

After the PCR finishes take Tube 1 - the 25μL vector PCR - and add 5μL Dpn1 (10U). Incubate this reaction overnight at 37°C in the PCR machine.

DpnI is active in the PCR buffer –and will digest any original template pCR4-GFP DNA that is present in this PCR reaction - note 5 μL/25 needs to be loaded on agarose gel later

**Remember to transfer the digests in Tubes 3 and 4 to the PCR machine at 37°C.**

**Day 2**

**6. Bacterial Transformations**

Label a new 1.5 ml tube T1 and add your initials.

To this add 5 μL from PCR tube 1 and 5 μL from PCR tube 2 by pipetting.

(contains DpnI cut PCR vector and mCherry PCR product) - mix gently by pipetting up and down and leave on ice for 15 mins.

Label three new 1.5ml tubes T2, T3 and T4 and add your initials.

To T2 pipette in 10 μL H_2_O

To T3 pipette in 10 μL from tube 3 above = +DNA -pCR4-EGFP digested

To T4 pipette 10 μL from tube 4 above = + DNA pCR4-EGFP undigested

**Add 100μL of competent cells to tubes T1, T2, T3 and T4 and mix gently then incubate them on ice for 30 mins.** to allow the DNA to adhere to the cells

Note transformation efficiency by competent cells is affected by temperature so **keep it on ice at all times**. Competent cells are bacteria whose membranes have been made permeable with various salts, allowing DNA to enter when they are ‘heat shocked’: briefly warmed to 42 ⁰C, and then cooled.

**7. Check the PCR products and DpnI digestions on an agarose gel**

You should have ~20 μL remaining in each of the PCR tubes 1 and 2, and 10 μL left in each of the DpnI reaction tubes 3 and 4 (pCR4-GFP DpnI treated and untreated).

Add 5 ul of 5x loading dye to PCR tubes 1 and 2.

Add 2.5 μl 5x loading dye to enzyme digestion tubes 3 and 4.

**Load the 10μL from each tube onto an agarose gel** alongside 5 ul of marker DNA that allows you to determine size of the DNA fragments.

Run the agarose gel for 45 mins at 115 V constant voltage

Take a photo of your agarose gel with resolved DNA bands and later stick it into this booklet.

**8.Transformation.**

Heat Shock: to allow DNA entry take T1-T4 containing the competent cells and place at 42°C for 2 mins

Then place the tubes on ice 5 mins.

Bacterial recovery: Add 100 μL LBroth at 37°C for 30mins

Resuspend cells by gently mixing and then pipette the 200 μL onto the LB-agar amp plates making sure that the cells are well spread with the spreader.

Ask Demonstrators for help with the spreading- and give the spread plates (labelled with your initials) to them for incubation in a 37°C incubator overnight.

| **Tube** | **Expected outcome** |
| --- | --- |
| T1 Digested template and Vector plus fragment |  |
| T2 no DNA |  |
| T3 template digested |  |
| T4 template undigested |  |

Fill out the expected outcome of the transformation in the table above.

Visualise your agarose gel in the gel dock system and add a photo of your gel here.

**Day 3**

Visualisation of the colonies from your experiment.

Photograph the plates and count the colonies. Record the number of colonies that are green and that are red for your own group and that of the whole cohort.

| **Tube** | **Actual outcome** | **Group outcome** |
| --- | --- | --- |
| T1 Digested template and Vector plus fragment |  |  |
| T2 no DNA |  |  |
| T3 template digested |  |  |
| T4 template undigested |  |  |

**Designing Primers:**

- Consider how much overlap is required with the vector and how much with the fragment
- GC content not too high or elongation times will be long or polymerase will not function
- Annealing temperature should be similar for PCRs.

**Table of PCR primer sequences.** Sequences of the primers used for PCR amplification of the pCR4 vector backbone and mCherry coding sequence, respectively. The primers for mCherry have 5’ 25 bp extensions (underlined) which do not anneal to the mCherry coding sequence. Instead, these 25 bp extensions are homologous to the ends of the linear pCR4 backbone PCR product, and are intended to facilitate recombination *in vivo*.

| Primer name | Sequence (5’- -3’) |
| --- | --- |
| pCR4 Backbone Forward | CGGCCGCTAAATTCAATTCG |
| pCR4 Backbone Reverse | GTTTAAACCTGCAGGACTAGTCC |
| mCherry Forward (25 bp homology arms to pCR4) | AGGGACTAGTCCTGCAGGTTTAAACGTGAGCAAGGGCGAGG |
| mCherry Reverse (25 bp homology arms to pCR4) | TAGGGCGAATTGAATTTAGCGGCCGTTCATTACTTGTACAGCTCGTCC |
